# Risk Factors for Accidents and Close Calls in Junior Freeriders, Adolescent Alpine Skiers and Adult Freeriders—A Comparison

**DOI:** 10.3390/ijerph192215076

**Published:** 2022-11-16

**Authors:** Anika Frühauf, Martin Kopp, Martin Niedermeier

**Affiliations:** Department of Sport Science, University of Innsbruck, 6020 Innsbruck, Austria

**Keywords:** self-objectification, youth sports, adventure sports, risk-taking behavior, gender, adolescence, ski racing

## Abstract

Understanding factors associated with risk-taking behavior, accidents and close calls could enhance prevention strategies and thus contribute to preventing serious injury or death in the long term. The following study aims to assess these factors in junior freeride athletes in comparison with competitive alpine skiers of similar age and adult freeriders. A cross-sectional questionnaire design was used to assess risk-related variables and potential associated factors. Accident and close call involvement did not significantly differ between the groups (*p* > 0.080). No significant relationships between gender and risk-related variables were found (|r| < 0.26). Precautionary behavior was higher in freeride adults and freeride juniors compared to alpine skiers (*p* < 0.001) and deliberate risk-taking was lower in freeride adults compared to junior freeriders and alpine skiers (*p* < 0.001). Regression analyses revealed that the association between self-objectification and accidents was stronger in freeride juniors compared to alpine skiers of similar age and freeride adults (*p* < 0.049). Although accidents and close calls were similar between groups, age seems to be an associated variable to deliberate risk-taking and precautionary behavior. The relationship between accidents and self-objectification in freeride juniors implicates a need for risk education in freeriders in the sensitive phase of adolescence beyond the mere presentation of environmental dangers.

## 1. Introduction

Alpine skiing is a popular sport, with an estimated number of 400 million skier days annually providing potential health benefits in the areas of psychosocial, cardiovascular, musculoskeletal and motor control [1]. However, as in most sport activities, skiing also inherits the risk of injuries [2]. Mortality in alpine skiing and snowboarding is higher compared to cross-country skiing (0.77 deaths per 1 million exposure days in alpine skiing and snowboarding vs. to 0.26 in cross-country skiing) [3]. However, mortality is still lower compared to alpine ski touring in the backcountry with an estimated mortality rate of 4.4 deaths per 1 million exposure days [3]. Whereas, in alpine skiing, non-traumatic (mostly cardiac) death is named as the main cause of mortality; avalanche-related burial resulting in trauma-related death is named as the main cause in alpine ski touring [3]. Freeride skiing and snowboarding, which encompasses riding off-piste in natural spaces outside the secured skiing resort, involves the danger of avalanche-related burial as well as further environmental factors (e.g., crevasses) which can result in serious injury or even death [4]. However, freeride skiing and snowboarding, which can involve alpine ski touring but also involves access using the lifts in the ski resorts, has largely increased within the last decades [5]. Competitions in freeriding have grown to over 130 events in several countries worldwide. The freeride junior tour, which includes freeriders up to 18 years, covers approximately half of these competitions and results in an annual junior world championship [6]. Since most studies on backcountry accidents retrospectively analyzed fatal avalanche accidents [7,8] data acquisition is limited to objective parameters (e.g., equipment, sociodemographic risk factors or environmental factors); more and more researchers call for an expansion towards analyzing close calls which come close to an accident but do not result in an accident [9]. This might be especially important in mountain sports activities [10]. Since close calls (also known as near misses in the literature [10]) are more frequent and less harmful than accidents, understanding factors associated with close calls could enhance prevention strategies and thus contribute to preventing serious injury or death in the long term [10].

The time of adolescence is a sensitive time of developmental changes and growth with a heightened need for reward sensitivity which is often linked to increased risk-taking [11,12]. Many behaviors, such as sport-related activities started during this time are more likely to be maintained throughout adulthood [13,14]. However, physical activity is low among adolescents; 81% of adolescent students worldwide (11–17 years) do not reach the recommended amount of physical activity with a higher inactivity in girls (84.7%) than in boys (77.6%) [15]. Many adolescents drop out of organized sports due to a lack of enthusiasm, as well as a lack of time and conflicts of interest [16,17]. Adventure sports, also known as high-risk sports or action sports, are mostly self-organized and do not fit the traditional pattern of club sports [18,19]. These include, for example, rock climbing, skateboarding, and freestyle and freeride skiing/snowboarding. In particular, young people aged 10 to 24 years, seem to show a high interest in these sports [20,21]. Therefore, adventure sports may help to decrease the high rate of adolescents showing insufficient physical activity. Furthermore, adventure sports encompass many aspects which are beneficial for healthy psychological development in adolescents, such as the need for autonomy, competence and relatedness, positive affect and self-efficacy and a possibility for positive risk-taking [11,12,22]. However, given the inherent risk of some of these activities [7], it is important to analyze factors associated with accidents to minimize the injury rates in adventure sports. These factors might be different from adult populations participating in adventure sports [23] considering the developmental changes connected to the time of adolescence [11].

Growing media interest, combined with improved technologies in sports equipment, could also be a contributing factor for a shift in participation away from traditional, organized club sports toward adventure-linked sports that involve some degree of subjective or objective risk [21]. The world of adolescents is changing rapidly, especially through technological innovations and the rise of social media [11]. Although social media provides opportunities for social learning, it also bears potential negative consequences, especially when interacting with picture-related social media activities [24]. Adolescents use social comparisons to develop self-identity which, nowadays, often takes place in a virtual setting [25]. Self-objectification is associated with social comparison and describes the individual’s awareness of how their body is perceived by others [26]. Objectification theory originates from a female perspective and provides the framework of how females are perceived through an objectifying (male) gaze, resulting in viewing one’s body as an object [27]. In Australia, young women experience an objectifying event every second day [28]. In recent years, self-objectification became a topic more researched in males [26,29]. Self-objectification was further shown to have negative effects on performance [30]. Social media is a tool that is especially important in the adventure sports industry; for the professionalization of adventure sports, athletes use social media to build up their fanbases and attract sponsorship by posting pictures or videos presenting themselves in their sport and associated lifestyle [21,31]. Having a sponsorship was named as a possibility to make a living through the sport by freeriders [5]. Thus, self-objectification might be a factor especially important in adolescents participating in adventure sports; however, to the best of our knowledge, self-objectification has not yet been researched in the context of risk-taking behavior.

Following these considerations, there seems to be a lack of knowledge in the accident-related variable of near misses in adventure sports, especially in the sensitive phase of adolescence, and in the role of self-objectification in the context of risk-related variables. Therefore, the present study aimed to assess associated factors with accidents and close calls as well as risk-related variables in adolescent adventure sports participants (freeride juniors) in comparison to two control groups: alpine skiers with a similar age and freeride adults. The group of alpine skiers allows a comparison to a sport which does not share the characteristics of adventure sports (e.g., risk in competitions). The group of freeride adults allows for analyzing risk-related factors connected to age since adult freeriders share the same sport but are not in the sensitive phase of adolescence.

## 2. Materials and Methods

### 2.1. Design and Procedure

The following study used a cross-sectional study design. Participants filled out a web-based questionnaire either by sending a link on their personal devices or through a provided tablet. Recruitment and data collection time points were dependent on the groups. Freeride juniors were part of the Freeride Junior World Championship; freeride adults were part of the Freeride World Qualifying Series. Participants of both groups were asked to fill out the questionnaire on a tablet when arriving at the registration. Alpine skiers were recruited through a ski-focused higher education school (Skigymnasium) which is a combination of school and professional ski training and through further e-mail recruitment in ski clubs. Answering the questionnaire was voluntary; participants could withdraw at any time in the process. The mean time to complete the questionnaire was 13.5 (SD: 4.4) minutes. The study conformed to the guidelines for conducting surveys which were approved by the Board for Ethical Questions in Science of the University of Innsbruck (#25/2016; #44/2021).

### 2.2. Participants

The total sample consisted of *n* = 144 participants with 39.9% (55) being female. The majority of the sample (55.6%, *n* = 80) was from Austria followed by the United States (12.5%, *n* = 18), and Switzerland (7.6%, *n* = 11). The distribution by the group was 45.1% (65) alpine skiers, 29.2% (42) freeride juniors and 25.7% (37) freeride adults. The median time practicing the sport was 5.5 (3.0–8.0) years for alpine skiers, 7.0 (4.3–10.0) for freeride juniors and 10.0 (6.0–13.0) for freeride adults. The inclusion criteria were age below 19 years for freeride juniors and alpine skiers and active participation in competitions for all three groups

### 2.3. Measurements

#### 2.3.1. Accidents and Close Calls

Accidents and Close Calls were assessed with the German version of the Accidents and Close Calls in Sports Inventory (G-ACCSI [32]). Close Calls were defined as “incidents that come very close to resulting in a negative outcome but that fail to materialize in a negative outcome” ([33], p. 480). The G-ACCSI is identical to the original ACCSI [9] in terms of factor structure and number of items [32]. The ACCSI was especially developed to provide an instrument which is not limited to the recall of accidents resulting in injuries but can further provide phenomenological data [9]. It consists of six items measuring accident involvement (three items; e.g., “I am involved in accidents when participating in my sport”) and experienced close calls (three items; e.g., “I find myself in situations that lead to near misses.”) in the activity. Participants responded to each item on a seven-point Likert scale from one (completely disagree) to seven (completely agree). Higher scores indicate higher accident/close-call involvement. The scale was used previously to compare slope skiers with freeride skiers [34]. Cronbach’s Alpha was 0.80 for accidents and 0.60 for close calls in the present sample.

#### 2.3.2. Risk-Taking Behavior

Risk-taking behavior was assessed using the German version of the Risk-Taking Inventory (G-RTI [32]). The G-RTI consists of three items measuring deliberate risk-taking (e.g., “I actively seek out dangerous situations”) and four items assessing precautionary behavior (e.g., “I take time to check for potential hazards”). Participants responded on a five-point Likert-scale ranging from one (never) to seven (always). The factor structure and number of items are identical to the original scale [33]. Cronbach’s Alpha was 0.74 for deliberate risk-taking and 0.80 for precautionary behavior in the present sample.

#### 2.3.3. Self-Objectification

Self-objectification was assessed using the Trait Self-objectification Questionnaire (TSOQ; [35]). The TSOQ consists of 10 attributes which are either listed as appearance-based (physical attractiveness, weight, sex appeal, measurements and muscle tone) or competence-based (strength, physical coordination, stamina/energy level, health, physical fitness). Participants are asked to rank the attributes in ascending order on how important each attribute is to their physical self-concept. Scores are computed separately for appearance and competence. The overall score ranges between −25 and +25 and reflects the difference between appearance and competence with a higher score indicating a higher self-objectification [35]. The TSOQ was used in male and female participants and demonstrates satisfactory validity with similar concepts of body image, appearance anxiety and body satisfaction [29,35]. Cronbach’s Alpha was 0.85 for appearance and 0.75 for competence in the present sample. We were unable to calculate self-objectification as a measure of internal consistency.

### 2.4. Statistical Analysis

The data were analyzed using jamovi (Version 2.3). The statistical analyses included tests on the differences between groups, correlational analyses and multiple regression analyses. As Shapiro–Wilk indicated a non-normal distribution of the data, Kruskal–Wallis tests were used to analyze the differences between alpine skiers, freeride juniors and freeride adults. Gender differences between groups were analyzed using χ^2^ tests. If significant group differences were found, Bonferroni-corrected post hoc tests were applied. As effects sizes rank biserial correlation *r* was used for metric variables and odds ratio (*OR*) for gender.

The correlational analyses between risk-related variables (accidents, close calls, deliberate risk-taking and precautionary behavior) and self-objectification, age and gender were conducted separately for each group to be able to detect group-specific characteristics in the associations. Spearman rank correlations were used for the metric variables and point-biserial correlation was used for risk-related variables and gender.

The primary aim of the regression analyses was to identify potential differences between groups in the role of self-objectification and the risk-related variables. Therefore, a series of multiple regression analyses were used to model each of the four risk-related variables (accidents, close calls, deliberate risk-taking and precautionary behavior) by group, age, self-objectification and the interaction between self-objectification and group. A significant interaction between self-objectification and group was interpreted as a group-specific differentiated correlation between self-objectification and the risk-related variable. Age was included in the model to control for age differences between groups. Gender was not included in the model since no significant group differences and no significant correlation was found to any of the risk-related variables. Assumption checks for the regression analyses included tests on multicollinearity using the variance inflation factor (all values below 1.99) and tests on the normality of the residuals (visual inspection of the Q-Q plots).

*p*-values < 0.05 were considered to indicate statistical significance (two-tailed) for all analyses. Unless otherwise stated, data is presented as Median (Mdn) and interquartile range (IQR) or relative (absolute) frequencies.

## 3. Results

### 3.1. Analysis on Group Differences

No significant group differences were found for the risk-related variables accidents and close calls (Table 1). Freeride adults reported significantly lower deliberate risk-taking and higher precautionary behavior compared to alpine skiers. Precautionary behavior was significantly higher in freeride adults compared to freeride juniors. Self-objectification was not significantly different between groups. Freeride adults were significantly older and showed a higher body mass. No significant gender differences were found.

### 3.2. Correlational Analysis by Group

The risk-related variables, accidents, close calls and deliberate risk-taking, showed positive correlations in all three groups (Table 2). Precautionary behavior was not significantly associated with the other risk-related variables in any of the three groups. Self-objectification was significantly positively related to accidents in freeride juniors, and non-significant in alpine skiers and freeride adults. This indicates a higher probability of accidents with higher values in self-objectification in freeride juniors only. Gender was not significantly associated with risk-related variables in any of the three groups. Age was significantly negatively associated with deliberate risk-taking in freeride adults, indicating a lower deliberate risk-taking in older adults.

**Figure 1:**
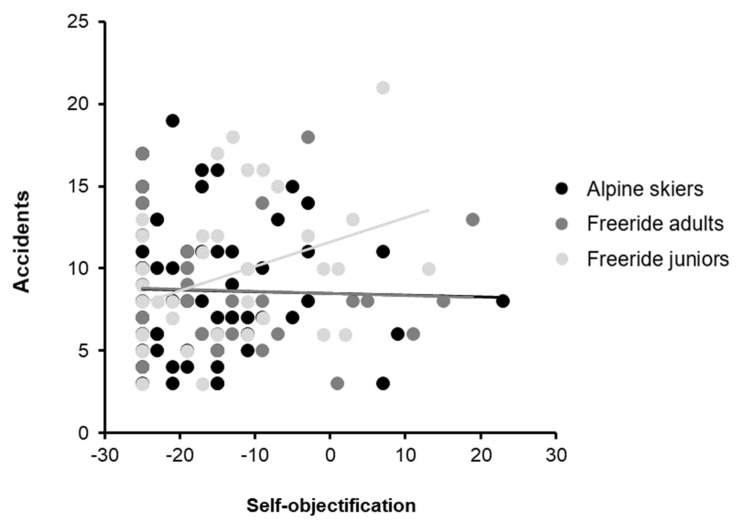
Group differences in the relation between self-objectification and accidents. The dots indicate values of each participant, the lines indicate the regression lines for each group.

The difference in the relation between self-objectification and accidents between groups is shown in Figure 1, where the regression lines from alpine skiers and freeride adults show a similar slope; whereas, freeride juniors show a different association between the two variables. In the junior class, freeriders with a higher self-objectification reported higher values in the accident subscale.

### 3.3. Multiple Regression Analyses

The results of the four separate regression analyses modeling the risk-related variables are shown in Table 3. The interaction between the groups and self-objectification was significant for the dependent variable accidents. Accordingly, freeride juniors showed a positive association between self-objectification and accidents indicating that a higher self-objectification was associated with a higher score in the subscale accidents. This significant relationship was not present for alpine skiers and freeride adults. Alpine skiers and freeride adults did not differ significantly. No significant interactions between group and self-objectification were found for the other dependent variables.

## 4. Discussion

The following study aimed to assess associated factors with risk-related variables in adolescent freeride athletes by comparing them with adult freeriders and adolescent alpine skiers. The following three main results were obtained: (1) regarding the between-group analysis, accident and close-call involvement were similar across the groups, deliberate risk-taking was lower and precautionary behavior was higher in adult freeriders; (2) no gender differences were found in any of the risk-related variables analyzed; and (3) the association between self-objectification and accidents was present in freeride juniors only, whereas both control groups did not show a self-objectification-accidents relationship.

### 4.1. Group Differences

The missing significant differences in accidents and close-call involvement for both the comparison of freeride juniors with alpine skiers as well as with freeride adults is not congruent with the literature. In a recent study, freeriders, both recreational and professional, experienced more accidents and close calls than recreational slope skiers [34]. One reason for this unexpected finding might be found in the fact that the present study assessed competitive athletes. Ski racing is an Olympic winter sport which is considered to have an above-average injury risk with almost half of the injuries occurring at official competitions [36]. This might have accounted for an elevated rate of accidents and close calls in alpine skiers and, ultimately, for a similar rate across all groups of the present study. Although no significant group differences were found in the present study for accidents and close calls, some risk-related variables were different between the groups. Deliberate risk-taking was lower in freeride adults compared to freeride juniors which indicates that age might be a decisive factor in risk-taking behavior in freeriding. Similar results were seen for precautionary behavior which was higher in freeride adults but also higher in freeride juniors compared to adolescent alpine skiers. Therefore, the type of activity seems to be relevant for risk-taking behavior. Although the traditional explanation for adventure sports participation was associated with a need or enjoyment to take risks, the present study is in line with multiple qualitative research results which found numerous descriptions of a conscious risk-taking behavior [5,22,37]. This might lead to a higher consciousness of risks in freeriders compared to alpine skiers as can be concluded from the results of the present study and previous descriptions in the literature [5].

When looking at quantitative comparisons on the influence of age on risk-taking behavior in the existing literature, no age differences were found in decision-making using a gambling task design in 8–30 years old individuals [38]. In a meta-analysis, risk-taking by description did not differ but risk-taking by learning [39]. This might be comparable to adult freeriders who have more experience and learned about risks. In a qualitative study, adult freeriders explained that they learned about risks through being a witness or involved, whereas young freeriders explained that they learned about risks through education [23]. This education of freeriders might have led to a higher precautionary behavior than in alpine skiers. Although wrong decisions in freeriding are potentially life-threatening [4], the fatality rate is low in ski racing with most accidents resulting in knee injuries [39]. Freeriders were shown to have a higher agency, the awareness to be able to intentionally influence one’s life, before and after the activity compared to slope skiers [34]. In competitions, freeriders can adapt their route choices and style of riding, whereas alpine skiers have no influence on route choices and marginal influence on the style of riding. The missing agency might explain the lower precautionary behavior in alpine skiers.

### 4.2. Gender in Risk-Related Variables

Although gender was a differing factor in injury occurrence in alpine skiers, with males being at a higher risk for injuries than females in a previous study [36], the present study did not find relevant relationships between gender and risk-related variables. In the backcountry, most fatal accidents occur due to avalanches, with over 75% of victims being male [7]. This might be partially accounted for by a higher participation rate of males [40]. However, multiple studies have shown differences in risk-taking behavior between males and females with males taking higher risks than females [38,41]. Sensation-seeking, which was long seen as the panoptic explanation for participation in risk-taking activities, was repeatedly found higher in males than in females throughout the last decades [42]. Although gender differences remained constant on most subscales of the sensation-seeking scale, gender differences in thrill and adventure-seeking declined, though men still scored higher than women [42]. Taking the criticism of sensation seeking as a measurement for participation in adventure sports into account [34,43], the thrill and adventure-seeking scale includes many aspects of the willingness to participate in thrill-enhancing activities [42]. Thus, the decline in gender differences could also account for a higher participation rate of women in adventure sports. The present study did not find any relationships between gender and deliberate risk-taking or precautionary behavior. The non-occurrence of gender differences might be due to a high experience in the specific sport activity in all groups and genders of the present study participants. This was similarly shown in experienced female mountain guides where no gender differences in actual behavior were reported although female mountain guides were perceived as having a lower risk tolerance [44].

### 4.3. The Role of Self-Objectification in Accidents

A new finding of the present study is the role of self-objectification in accidents. While relatively unrelated in alpine skiers and adult freeriders, it seems to be a relevant variable for accident involvement in junior freeriders. In freeride juniors, those who have a higher self-objectification, thus focusing more on appearance, a higher rate of accidents is reported. This seems to be unique for junior freeriders as this was not present in older freeriders and same-aged alpine skiers. Self-objectification was shown to have negative effects on performance in cognitive tasks [30]. Freeriding is a complex activity in a dynamic environment involving physical and cognitive abilities [4]; however, since the relation between self-objectification and accidents was only significant in freeride juniors, the activity, per se, might not be a valid explanation for the relationship of self-objectification and accidents. Since this difference could be seen in freeride juniors only, the explanation should focus on the combination of activity (freeriding) and age (adolescence). Unlike alpine skiing where you have the possibility to make a living through good rankings in competitions, the prize money in freeriding is not sufficient to make a living. Sponsorship was named as a possibility to make a living through the sport [5,45]. Sponsorship can be gained and maintained through a good self-representation on social media or through picture and film involvement which subsequently can clear the path for a career in adventure sports [31,45]. In an experimental laboratory study, consuming risk-promoting media was shown to lead to higher risk-taking [46]. However, this relationship was not investigated in adventure sports or outside the laboratory yet. Although the importance of social media self-promotion in professionalizing adventure sports and marketing brands is widely recognized in the literature, no link to risk-taking or accidents has been established yet [45,47,48]. Furthermore, the time of adolescence is associated with an increased need for risk-taking [11], which might result in a higher propensity for risk-taking in picture-related activities. Thus, coaches should be aware of the role of self-objectification in accident involvement in freeriding, by raising awareness of the risks associated with self-representation in adolescents’ social media activity. Future research should consider self-objectification and potential relations to social-media use and risk-taking behavior in the sensitive age of 16 to 18 years in junior freeriders.

### 4.4. Limitations

When interpreting the results, the following limitations should be taken into account. The present sample is solely based on competitive athletes; thus, the results should not be generalized to slope skiing and freeriding in general. Due to the limited number of athletes available, the sample size is rather low. It would be interesting to investigate also recreational skiers and freeriders. It should be noted that Cronbach’s alpha for the subscale Close Calls was rather low (0.60) in the present sample, the internal consistency of the scale should be interpreted cautiously. Furthermore, no causal links can be drawn based on a cross-sectional study. Cross-sectional studies, in general, bear the limitation of a potential recall bias or non-truthful answered questions due to social desirability.

## 5. Conclusions

Unlike most previous quantitative research on the population of freeriders or, more broadly, adventure sports participants, the present study did not find relevant relationships between gender and risk-related variables. Age seems to be a decisive factor in risk-taking behavior, as freeride adults reported the highest precautionary behavior scores and lowest risk-taking. Since precautionary behavior was also higher in freeride juniors, compared to alpine skiers, the group of freeriders might have a higher awareness of risks. This difference might be connected to a higher possibility to influence route choices through decision-making in freeriding, which is only marginally present in competitive alpine skiers due to fixed route settings. The association between self-objectification and accidents was higher in freeride juniors compared to both control groups which indicates that the time of adolescence, as well as the activity of freeriding, may be decisive factors in this relationship. Since athletes in adventure sports are dependent on sponsorships to make a living out of their sport, self-presentation could have a higher importance, which might add to a higher risk of impressive pictures resulting in more accidents in those who focus more on appearance. Coaches and competition managers should be aware of this relationship to be able to raise awareness in adolescents. Future studies should further investigate the new finding of the role of self-objectification in accidents on its robustness by investigating adolescent freeriders in particular and adventure sport participants, in general, using larger sample sizes.

## Figures and Tables

**Table 1:** Data on risk-related variables, self-objectification and demographic aspects by group.

	Alpine Skiers (*n* = 65)		Freeride Juniors (*n* = 42)		Freeride Adults (*n* = 37)		Inferential Statistics			Effect Sizes		
	Med	(IQR)	Med	(IQR)	Med	(IQR)	*χ*^2^ (2, *N* > 135)	*p*	post hoc	r AS:FJ	r FJ:FA	r AS:FA
Accidents (3: low, 21: high)	8.0	(6.0–11.0)	8.0	(6.0–12.0)	8.0	(6.0–10.0)	0.45	0.800		0.06	0.08	00.01
Close calls (3: low, 21: high)	12.0	(9.0–13.0)	12.0	(10.3–15.0)	10.0	(8.0–12.0)	5.04	0.080		0.14	0.30	0.14
Deliberate risk-taking (3: low, 21: high)	9.0	(7.5–10.5)	9.0	(7.0–11.0)	6.0	(5.0–8.0)	16.93	**<0.001**	AS:FA, FJ:FA	0.02	**0.45**	**0.46**
Precautionary behavior (3: low, 21: high)	11.0	(10.0–15.0)	16.5	(14.0–18.0)	18.0	(17.0–20.0)	66.45	**<0.001**	AS:FA, AS: FJ, FJ:FA	**0.65**	**0.36**	**0.88**
Self-objectification (−25: low, 25: high) ^a^	−19.0	(−23.0–−13.0)	−15.0	(−24.0–−9.0)	−19.0	(−25.0–−9.0)	1.20	0.550		0.14	0.09	0.01
Appearance (15: low, 40: high) ^a^	13.0	(11.0–16.0)	15.0	(10.5–18.0)	13.0	(10.0–18.0)	0.99	0.608		0.12	0.08	0.01
Competence (15: low, 40: high) ^a^	32.0	(29.0–34.0)	30.0	(27.0–34.0)	31.5	(26.8–35.0)	1.66	0.436		0.16	0.09	0.04
Age, years	16.0	(16.0–18.0)	17.0	(16.0–18.0)	25.0	(21.0–30.0)	86.51	**<0.001**	AS:FA, FJ:FA	0.25	**1.00**	**1.00**
Body mass, kg ^a^	63.0	(57.8–68.3)	65.5	(60.0–70.5)	68.5	(63.0–75.0)	0.89	**0.012**	AS:FA	0.19	0.17	**0.36**
Body height, m ^a^	1.75	(1.70–1.78)	1.75	(1.68–1.82)	1.74	(1.70–1.80)	0.25	0.883		0.06	0.01	0.04
	**%**	**(*n*)**	**%**	**(*n*)**	**%**	**(*n*)**	***χ*^2^ (2, *N* = 138)**	***p***		**OR AS:FJ**	**OR FJ:FA**	**OR AS:FA**
Gender ^a^												
Female	43.1	(28)	38.5	(15)	35.3	(12)	0.61	0.738		0.83	0.87	0.72
Male	56.9	(37)	61.5	(24)	64.7	(22)						

^a^ Missing cases < 10, Med: Median, IQR: Interquartile range, AS: Alpine skiers, FJ: Freeride juniors, FA: Freeride adults, OR: odds ratio, bold values indicate significant differences between groups.

**Table 2:** Intercorrelations between risk-related variables, self-objectification and demographic aspects by group.

Variable		Alpine Skiers				Freeride Juniors				Freeride Adults			
		1	2	3	4	1	2	3	4	1	2	3	4
1	Accidents	-				-				-			
2	Close calls	**0.63**	-			0.20	-			**0.45**	-		
3	Deliberate risk-taking	0.09	**0.28**	-		0.24	**0.35**	-		**0.53**	**0.45**	-	
4	Precautionary behavior	−0.05	−0.06	0.12	-	0.24	0.19	0.20	-	−0.16	0.06	−0.12	-
5	Self-objectification	0.02	−0.10	−0.03	−0.19	**0.35**	0.19	0.21	−0.20	−0.14	0.07	0.08	−0.20
6	Age	0.15	0.01	0.23	0.10	−0.05	0.04	−0.23	−0.02	−0.29	−0.20	**−0.41**	0.15
7	Gender	0.14	0.08	0.09	−0.18	−0.05	−0.16	0.02	−0.02	0.26	0.24	−0.08	0.04

Values are Spearman rank correlation coefficients for all variables, except for gender, where point-biserial correlation coefficients were given. Bold values indicate significant correlation coefficients.

**Table 3:** Four separate regression analysis on the risk-related variables accidents, close calls, deliberate risk-taking and precautionary behavior as outcomes of group, self-objectification, age, and the self-objectification-by-group interaction.

Variable	Accidents				Close Calls				Deliberate Risk Taking				Precautionary Behavior			
	*p*	β	95% *CI* *lb*	95% *CI* *ub*	*p*	β	95% *CI* *lb*	95% *CI* *ub*	*p*	β	95% *CI* *lb*	95% *CI* *ub*	*p*	β	95% *CI* *lb*	95% *CI* *ub*
Intercept	**<0.001**				**<0.001**				**<0.001**				**<0.001**			
Group																
Freeride juniors–Alpine skiers	**0.026**	**0.21**	**−0.19**	**0.62**	**0.018**	**0.48**	**0.07**	**0.89**	0.100	0.01	−0.38	0.40	**<0.001**	**1.18**	**0.87**	**1.49**
Freeride adults–Alpine skiers	0.434	0.38	−0.35	1.10	0.160	0.13	−0.62	0.88	0.810	−0.42	−1.13	0.29	**<0.001**	**1.55**	**0.98**	**2.11**
Self-objectification	0.791	−0.04	−0.31	0.24	0.419	−0.11	−0.37	0.16	0.375	−0.11	−0.36	0.14	0.240	−0.12	−0.32	0.08
Age	0.222	−0.19	−0.50	0.12	0.361	−0.15	−0.46	0.17	0.170	−0.21	−0.51	0.09	0.870	0.02	−0.22	0.26
Self-objectification ∗ group																
Self-objectification ∗ (Freeride juniors–Alpine skiers)	**0.049**	**0.43**	**0.00**	**0.86**	0.216	0.26	−0.15	0.67	0.055	0.39	−0.01	0.78	0.808	0.04	−0.27	0.35
Self-objectification ∗ (Freeride adults–Alpine skiers)	0.936	−0.02	−0.41	0.38	0.065	0.39	−0.02	0.80	0.062	0.37	−0.02	0.76	0.558	0.09	−0.22	0.40

Accidents R^2^ = 0.06, *p* = 0.232, Close calls R^2^ = 0.10, *p* = 0.038, Deliberate risk-taking R^2^ = 0.19, *p* < 0.001, Precautionary behavior R^2^ = 0.49, *p* < 0.001, β: standardized regression coefficient, *CI*: confidence interval, *lb*: lower bound, *ub*: upper bound. Bold values indicate significant regression coefficients.

## Data Availability

Data is available upon reasonable request by the authors.
